# 

**Published:** 1872-04

**Authors:** 


### Contents of April Number.

### ORIGINAL DEPARTMENT.

ART. I.—Diptheritic Croup and its Treatment. A Paper Read before the Buffalo Medical Association, January 2d, 1872, by F. W. Bartlett, M. D. .......... 321

ART. II.—Medical Society of the County of Albany. Semi-Monthly Meeting, March 20th, 1872. Reported by James S. Bailey, M. D. 331

ART. III.—Abstract of the Proceedings of the Buffalo Medical Association, April 2d, 1872 .......... 336

ART. IV.—On the Recent Advances in the Theory of Vision. By H. Helmholtz. Translated by F. W. Abbott, M. D .......... 346

### CORRESPONDENCE.

Cerebro-Spinal Meningitis in Canada .......... 355

### EDITORIAL DEPARTMENT.

Cerebro-Spinal Meningitis, or "Spotted Fever," .......... 359

Calabar Bean in Spotted Fever .......... 361

American Medical Association .......... 361

Death of Dr. Zina Pitcher, of Detroit .......... 362

Cundurango by its Friends .......... 362

BOOKS REVIEWED:

A Treatise on Human Physiology; designed for the use of students and practitioners of medicine. By John C. Dalton, M. D. Fifth Edition .......... 363

Physiology of the Soul and Instinct, as distinguished from Materialism. By Martin Paine, A.M., M.D., LLD. ... 363

Books and Pamphlets Received .......... 364

THE BUFFALO

